# Phenotypic and genomic comparison of dominant and nondominant sequence-type of *Acinetobacter baumannii* isolated in China

**DOI:** 10.3389/fcimb.2023.1118285

**Published:** 2023-02-20

**Authors:** Xiaoyang Kong, Tao Chen, Lihua Guo, Yanzi Zhou, Ping Lu, Yonghong Xiao

**Affiliations:** ^1^ State Key Laboratory for Diagnosis and Treatment of Infectious Diseases, National Clinical Research Center for Infectious Diseases, Collaborative Innovation Center for Diagnosis and Treatment of Infectious Diseases, The First Affiliated Hospital, Zhejiang University School of Medicine, Hangzhou, China; ^2^ Department of Rheumatology, Affiliated Hangzhou First People’s Hospital, School of Medicine, Zhejiang University, Hangzhou, China; ^3^ Jinan Microecological Biomedicine Shandong Laboratory, Jinan, China

**Keywords:** *Acinetobacter baumannii*, sequence type, resistance, virulence, transcriptome

## Abstract

*A. baumannii* is a common clinical pathogen that often causes pneumonia and bloodstream infections in ICU patients. Sequence types (ST) are used to investigate the distribution and spread of *A. baumannii*. Biological characteristics such as virulence and resistance may play a role in *A. baumannii* becoming a specific dominant ST(DST,ST191, ST195 and ST208) strain. To characterize the biological, genetic, and transcriptomic differences between the DST and non-dominant ST(NST,ST462 and ST547,etc.) strains in *A. baumannii*, we performed several biological experiments and genetic, and transcriptomic analyses. The DST group displayed more resistance ability to desiccation, oxidation, multiple antibiotics, and complement killing than the NST group. However, the latter had higher biofilm formation ability than the former. The genomic analysis showed the DST group exhibited more capsule-related and aminoglycoside-resistant genes. Besides, GO analysis indicated that functions involved in lipid biosynthetic, transport, and the metabolic process were up-regulated in the DST group, while KEGG analysis manifested that the two-component system related to potassium ion transport and pili were down-regulated. In short, resistance to desiccation, oxidation, multiple antibiotics, and serum complement killing are important reasons for the formation of DST. Genes related to capsule synthesis and lipid biosynthesis and metabolism play an important role at the molecular level in the formation of DST.

## 1 Introduction

*A. baumannii* is a Gram-negative bacterium that causes a number of clinically important, life-threatening infections including ventilator-associated pneumonia, bloodstream infections, and intracranial infections (Joly-Guillou, 2005; Maragakis and Perl, 2008; Freire et al., 2016). Furthermore, because of its inherent antibiotic resistance and the ease with which it acquires resistance elements from elsewhere to develop multidrug resistance, carbapenem-resistant *A. baumannii* has become a common clinical pathogen and is listed by WHO as a pathogenic bacterium requiring priority development of new antibacterial drugs (World Health Organization; Lee et al., 2017).

ST is often used to investigate the prevalence, transmission, and outbreaks of *A. baumannii* clones in different regions or hospitals, and thus to assist in formulating appropriate hospital infection control measures (Schultz et al., 2016). Several studies, in different regions of China, have shown that ST191, ST195, and ST208 (Oxford scheme) are the most prevalent *A. baumannii* ST types isolated (Deng et al., 2014; Xiao et al., 2016; Ning et al., 2017; Zhou et al., 2018). ST208 and ST191 accounted for 58.7% and 10.9%, respectively, of strains isolated in Shanghai, whereas 41.7% and 13.1% of the strains isolated in the south of China were ST195 and ST208, respectively. ST191 was also shown to be the most prevalent strain in Korea (Son et al., 2020). Consequently,we define ST191 ST195 and ST208 these top three dominant ST types as the DST, and the other ST types including ST462, ST547 and STn(new types of numbers not yet assigned) as non-dominant sequence type(NST).

Studies have shown that the outer membrane protein OmpA, phospholipase, capsule capsular polysaccharide(CPS), iron acquisition system, efflux pump, Csu chaperone usher-type pilus, and secretion system are all important virulence factors of *A. baumannii* (Harding et al., 2018). Moreover, the ability to survive in unfavorable environments has been suggested as an important virulence strategy. Indeed, it has been reported that specific ST strains are highly virulent and correlate with poor clinical prognosis in infected patients (Yoon et al., 2019).

Although desiccation resistance has been reported to contribute to the spread and persistence of type-specific *A. baumannii* in the hospital setting (Giannouli et al., 2013). The factors that facilitate the generation of DST, and the phenotypic and genotypic differences between DST and NST strains of *A. baumannii*, have not been fully elucidated. We combined biological experiments with genomic and transcriptomic analysis, therefore, to comprehensively elucidate the biological properties, especially those relating to virulence and resistance, that differentiate DST and NST strains.

## 2 Materials and methods

### 2.1 Isolates, culture conditions, and susceptibility tests

All *A. baumannii* strains were isolated from a tertiary, first-class teaching hospital in East China, and the strain number, ST type, etc. are shown in **Table S1**. 10 strains of ST191, 12 strains of ST195, 11 strains of ST208, 10 strains of ST462, and 9 strains of ST547, total 52 strains were used for each biological experiments. Two strains from each ST type above, plus two STn strains, a total of 12 strains were selected for subsequent genomic and transcriptomic analysis based on their ability to resist complement killing. The antimicrobial susceptibility of all *A. baumannii* isolates was determined using agar dilution according to the Clinical and Laboratory Standards Institute (CLSI) guidelines from 2020. Resistance breakpoints determined by the European Committee on Antimicrobial Susceptibility Testing (EUCAST) were used for polymyxin B.

### 2.2 Growth curves

Growth curves were performed as described previously with slight modifications (Hall et al., 2014) Briefly, A. baumannii in the log-phase of growth were adjusted to 0.5 absorbance units (OD600) and diluted 50-fold with LB medium. Aliquots (200 μl) were added to a 96-well plate, placed in a microplate reader at 37°C, and shaken for 5 s every 30 min, and the OD600 was measured for 24 h.

### 2.3 Desiccation resistance assays

Desiccation resistance assays were completed as previously described (Boll et al., 2015) Log-phase A. baumannii were harvested and washed twice with an equal volume of LB medium. Each sample was adjusted to 1×108 CFU/ml and then serially diluted and plated to determine the input CFU. Bacterial suspension (10 μl) was spotted onto 96-well polystyrene plates and desiccated at 30°C at a humidity of 40%. PBS (200 μl) was added to each well to resuspend the bacteria after 48 h and 96 h, respectively, and the resuspended bacteria were serially diluted and plated to calculate the output CFU. The percent of survival was defined as the ratio of output to input CFUs.

### 2.4 Oxidative killing assay

*A. baumannii* in the log-phase of growth were adjusted to OD_600_ = 0.5 and spread evenly on an MH agar plate. A sterile filter paper disk with a diameter of 6 mm was placed in the middle of the inoculated agar, and 10 μl 20% (v/v) hydrogen peroxide was pipetted onto the filter paper. The plates were incubated at 37°C for 18 h, after which the growth inhibition zone around the disk was measured using Vernier calipers.

### 2.5 Antiserum complement killing

Blood collected from healthy volunteers was centrifuged and filtered with a 0.22 μm pore size syringe filter to obtain sterilized serum. Half of the serum was inactivated by heating in a 56°C water bath for 30 min. *A. baumannii* in the log-phase of growth were adjusted to 2 × 10^6^ CFU/ml and mixed with normal or inactivated serum at a ratio of 1:9, respectively. After incubation for 1 h at 37°C, samples were serially diluted and spread on MH agar plates. After overnight incubation at 37°C, bacteria colonies were counted, and the bacterial survival rate was calculated by the following formula: Bacterial survival rate = (number of colonies in normal serum/number of colonies in inactivated serum) × 100%.

### 2.6 Biofilm formation

*A. baumannii* in the log-phase of growth were adjusted to 0.5 absorbance units (OD_600_), before 200 μl aliquots were pipetted into the wells of a 96-well plate. Bacteria were cultivated in an incubator for 24 h at 37°C and 5% CO_2_, to allow biofilm formation, before nonattached bacteria were removed from the wells by aspiration. Biofilms were gently washed with ddH_2_O, after which the 96-well plate was inverted on absorbent paper for 15 min at room temperature. Biofilm cells were fixed by adding 4% (v/v) paraformaldehyde to each well for 20 min and were then stained with 1% (w/v) crystal violet for 15 min. Biofilms were washed thoroughly with ddH_2_O, before the bound crystal violet was eluted with 200 μl 95% ethanol, and were then incubated at 37°C for 30 min. The crystal violet eluted from each biofilm was measured colorimetrically at OD_570_.

### 2.7 G. mellonella larvae infection

*A. baumannii* in the log-phase were adjusted to 1× 10^7^ CFU/ml. *G. mellonella* larvae weighing ~300 mg was divided randomly into groups, with each group containing 15 larvae. Each larva was infected with 10 μl of the adjusted bacterial suspension, incubated at 37°C, and observed once every 12 h for 3 days. Larval survival status was assessed using the acupuncture method, and no response was considered as death.

### 2.8 Whole genome sequencing and genome analysis

*A. baumannii* genomes were extracted using QIAamp DNA Mini Kit (QIAGEN, Valencia, CA), whole genome sequencing was performed by Illumina HiSeq 2500 (Illumina, San Diego, CA), and the raw sequencing results were quality-controlled using FastQC v.0.11.5. Trimmomatic v.0.40 was used to trim splice regions. The trimmed reads were assembled and annotated by SPAdes(http://cab.spbu.ru/software/spades/) v.3.6 and RAST(https://rast.nmpdr.org/), respectively. Antibiotic resistance genes and virulence genes were identified using the ResFinder (Bortolaia et al., 2020) and VFDB databases (Chen et al., 2016), respectively, with 80% identity and 80% query coverage as the cutoff values.

### 2.9 RNA sequencing and quality control

*A. baumannii* RNA library preparation, construction, sequencing, and processing of reads were performed at the Novogene Co., Ltd**. (**Beijing, China**)**. The raw data were first processed by a Perl script to remove reads with adapters, those containing poly-N and low-quality reads. The sequencing error rate for a single base position should be less than 1% and no more than 6% (**Table S2**). All the analyses below were based on high-quality clean data.

### 2.10 Quantification of gene expression levels and analysis of differentially expressed genes (DEGs)

Clean reads were mapped to *A. baumannii* MDR-ZJ06 (accession No. CP001937.2) using Bowtie2-2.2.3. Counting the reads numbers mapped to each gene was achieved using HTSeq v0.6.1. Differential expression analysis for the DST and NST groups was performed using the DESeq R package (1.18.0), and *p*-values were corrected using the Benjamini–Hochberg method to control for false discovery rate (Glickman et al., 2014). Genes for which corrected *p*-values <0.05 were obtained, after DESeq processing, were defined as differentially expressed.

### 2.11 GO, COG, and KEGG enrichment analysis

Gene ontology (GO) enrichment analysis of DEGs was performed using the GOseq R package. GO terms with *p*-values less than 0.05 were considered significantly enriched for DEGs. Subsequently, the COG database was used to identify the functions of the proteins encoded by the DEGs. KOBAS software was used to test the statistical enrichment of DEGs in the KEGG pathway.

### 2.12 Quantitative real-time PCR

For analysis of expression of specific genes, *A. baumannii* RNA was first stabilized with RNA protection solution (Qiagen, 74124) and then extracted and purified according to the instructions of the RNeasy Mini Kit (Qiagen, 74104). One microliter RNA was added to nine microliter reaction buffer from the PrimeScript™ RT reagent Kit (Takara, RR037A) in the appropriate ratio, and then reverse transcribed to generate cDNA as follows: 37°C for 15 min, 85°C for 5 sec, and 4°C. cDNA was added to the reaction system of the TB Green^®^ Premix Ex Taq™ (Takara, 420A), and PCR was carried out as follows: 95°C, 30 s for one cycle (stage 1); 95°C, 5 s, 60°C, 34 s for 40 cycles (stage 2); and 95°C, 15 s, 60°C,1 min, 95°C 15 s (stage 3). The genes and primers used are shown in **Table S3**, and *recA* was selected as the internal reference. The relative expression of different genes was calculated by the 2^-ΔΔCt^ method.

### 2.13 Statistics

Statistics were performed using prism9. Two groups were compared using the Mann-Whitney t-test, and the five groups were compared using a one-way ANOVA, with horizontal lines representing means ± standard error of means; *p < 0.05, **p < 0.01, ***p < 0.001, **** *p* < 0.0001.The biological experiments were repeated at least three times, except for the growth curve experiment and anti-desiccation assay, where two biological replicates were done, and the biofilm experiment, where four biological replicates were done.

## 3 Results

### 3.1 Comparison of the biological functions of DST and NST groups

We first compared their growth and there was no difference in the growth rates of strains in the two groups (**Figure S1**, *p* = 0.42).

**Figure 1:**
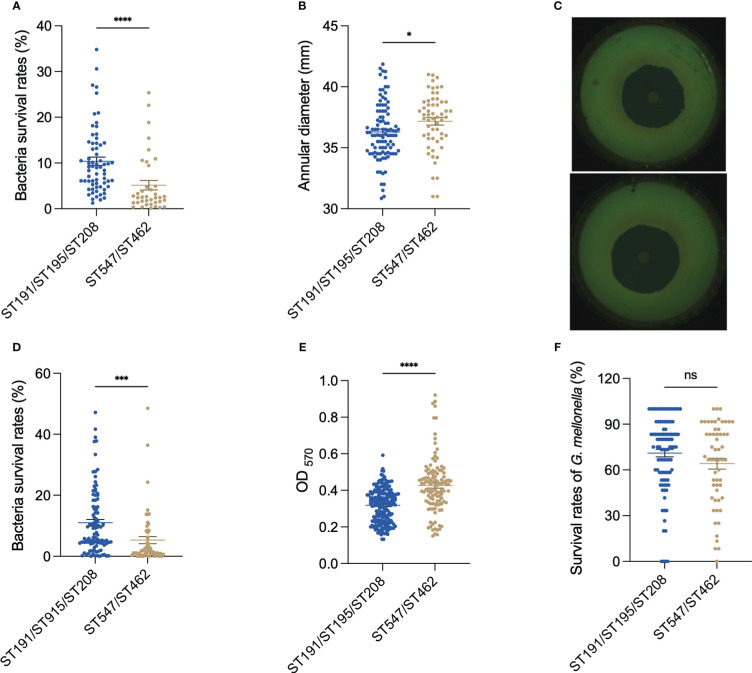
Comparison of (*A*) *baumannii* DST and NST group phenotypes. **(A)** Antidesiccation *in vitro.* **(B)** Antioxidation *in vitro*. **(C)** Representative pictures of the antioxidation *in vitro.* The upper figure is the inhibition circle of hydrogen peroxide in the DST group, while the lower is the inhibition circle in the NST group. **(D)** Antiserum killing *in vitro*. **(E)** Biofilm formation ability of bacteria *in vitro*. **(F)** The survival rate of G. mellonella after being infected with A. baumannii at 72 h. Each point represents data from one biological experiment for one bacterium. Each experiment included 10 strains of ST191, 12 strains of ST195, 11 strains of ST208, 10 strains of ST462, and 9 strains of ST547, and bar plots illustrate means ± SEM. Meaning of symbols “*, ***, **** and ns” indicate p<0.05, p<0.001, p<0.0001 and no statistical difference (p>0.05), respectively.

As resistance to desiccation and oxidative stress are essential for the persistence of *A. baumannii* in the medical environment, we compared their abilities in these two aspects. After 96 h of desiccation, strains in the DST group had a mean survival rate of 10.38%, while strains in the NST group had a mean survival rate of 5.13% (*p* < 0.0001; **Figure 1A**). Moreover, the oxidative stress analysis showed that the hydrogen-peroxide-induced growth inhibition zone for the DST group was lower than that for the NST group (zone diameter 36.29 mm vs. 37.16 mm; *p* =0.027), indicating that the DST strains were more resistant to oxidative stress than the NST strains (**Figures 1B, C**).

The DST group had higher resistance to complement-mediated killing (mean survival 11.00% vs. 5.31%; *p* < 0.0001; **Figure 1D**), but lower biofilm formation ability (OD_570_ 0.31 vs. 0.42; *p* < 0.0001), than the NST group (**Figure 1E**).

When *G. mellonella* was used as an *in vivo* bacterial infection model, larvae infected with the DST group had a higher mean survival rate than those infected with the NST group in the early (12 h), middle (36 h), and late (72 h) stages of infection, however, all this difference was not statistically significant (**Figures 1F**, **S2**).

### 3.2 Antimicrobial susceptibility

All strains belonging to the DST group were resistant to all three cephalosporins, while the strains of the NST group displayed resistance rates of 33.34%, 66.66%, and 33.34% to ceftazidime, cefepime, and ceftriaxone, respectively. Similarly, the DST strains were all resistant to carbapenems, whereas 66.66% of the NST group strains showed resistance. However, for aminoglycosides, the resistance rate was 66.66% for the DST group and 16.66–33.34% for the NST group. All the DST group strains were resistant to quinolones, while the NST group strains had a resistance rate of 66.66%. The overall MICs of the DST group were also higher than those of the ST group for minocycline and combined antibiotics (**Table 1**).

**Table 1:** Antibiotic susceptibility of *A. baumannii* DST and NST strains.

Class	ST	Strains	SCP	CZA	SAM	TZP	SXT	CAZ	FEP	CRO	IMP	MEM	GEN	AMK	CIP	LVX	MIN	POL
DST	191	15923	128/64	>32/4	64/32	>128/4	>8/152	128	32	>128	>64	>64	>128	>128	>32	16	8	0.5
	191	26128	128/64	>32/4	>128/64	>128/4	>8/152	128	>128	>128	>64	>64	8	4	>32	16	2	0.5
	195	16149	128/64	>32/4	128/64	>128/4	0.5/9.5	>128	64	>128	>64	>64	>128	>128	>32	8	4	0.5
	195	16354	>128/64	>32/4	>128/64	>128/4	>8/152	>128	128	>128	>64	>64	>128	>128	>32	8	4	0.25
	208	19701	128/64	>32/4	32/16	>128/4	>8/152	128	32	>128	>64	>64	>128	>128	>32	8	8	1
	208	17493	>128/64	>32/4	>128/64	>128/4	>8/152	128	128	>128	>64	>64	8	8	>32	16	8	1
NST	462	24443	128/64	>32/4	64/32	>128/4	<0.25/4.75	4	64	16	>64	>64	8	16	>32	8	<0.25	0.5
	462	19595	128/64	>32/4	32/16	>128/4	<0.25/4.75	4	32	16	64	>64	16	16	>32	8	<0.25	0.5
	547	18190	>128/64	>32/4	>128/64	>128/4	>8/152	>128	>128	>128	>64	>64	>128	>128	>32	8	4	0.25
	547	12134	128/64	>32/4	>128/64	>128/4	>8/152	>128	128	>128	>64	>64	8	8	>32	8	4	0.5
	New	6070	128/64	>32/4	16/8	16/4	<0.25/4.75	4	2	16	0.5	0.5	1	4	0.5	0.125	<0.25	1
	New	24484	128/64	4/4	8/4	16/4	<0.25/4.75	4	2	16	0.25	0.25	1	2	1	0.25	<0. 25	0.5

SCP, cefoperazone/sulbactam; CZA, ceftazidime/avibactam; SAM, ampicillin/sulbactam; TZP, piperacillin/tazobactam; SXT, cotrimoxazole; CAZ, ceftazidime; FEP, cefepime; CRO, ceftriaxone; IPM, imipenem; MEM, meropenem; GEN, gentamicin; AMK, amikacin. CIP, ciprofloxacin; LVX, levofloxacin; MIN, minocycline; POL, polymyxin B.

### 3.3 Virulence and resistance genes analysis based on genomes of DST group and NST group

The general features of the twelve genomes were shown in **Table S4**. The phylogenetic tree built using roary software (default parameters) indicates that strains of the same ST type were closely related, but ST208 of the DST group was evolutionarily distant from ST191 and ST195. ST547 of the NST group was also distant from ST462 and the STn type strains (**Figure S3**).

**Figure 2:**
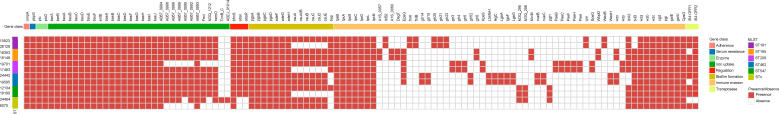
Comparison of virulence genes present in *A. baumannii* DST and NST strains. The first row represents the virulence gene name, and the second row represents gene functional classification. The columns from left to right represent the name and ST type. Both identity and coverage are greater than 80% as the threshold for the presence of genes.

The results appeared that the two groups shared significant virulence genes. The encoded products of which included adhesion-related outer membrane protein OmpA, serum resistance-associated penicillin-binding protein PbpG, cleavage cell membrane related phospholipase Plc and PlcD, iron acquisition protein BarAB, BauABCDE, AbaI and AbaR related to quorum sensing, PgaABCD involved in biofilm formation, the CPS synthesis component, Wza, Wzb and Wzc related to immune escape, etc (**Figure 2**). This is consistent with the similar virulence seen for strains in the two groups in the *G. mellonella* infection model.

Twenty-four virulence genes associated with CPS were found only in strains of the DST group. These include glycosyltransferase-related genes *itrB2*, *gtr3/4/5/8/20/21/22*, *kpsS*, CMP-glycan pathway-related *pseB/C/F/H/I*, UDP-glycan pathway-related *fnlA/fnlB*, dehydrogenase *pgt1*, and isomerase *wecB*. However, 17 virulence-associated genes were found exclusively in strains from the NST group. These include 15 genes related to the CPS (CMP-glycan pathway-related *legC*, *lgaA/F/G* gene, glycosyltransferase *gtr14/15/52*, *M3Q_295/296*, and *weeH*) and two genes related to iron acquisition.

Overall, strains of the DST group harbored a greater number of CPS synthesis-related genes than those of the NST group, consistent with the increased resistance to complement killing and desiccation previously reported for DST group strains (Russo et al., 2010; Tipton et al., 2018). Many different genes related to the K locus, which is associated with CPS synthesis in *A. baumannii*, were unique to members of each of the two groups, consistent with the high variability previously reported for the middle region at the K locus (Kenyon and Hall, 2013).

**Figure 3:**
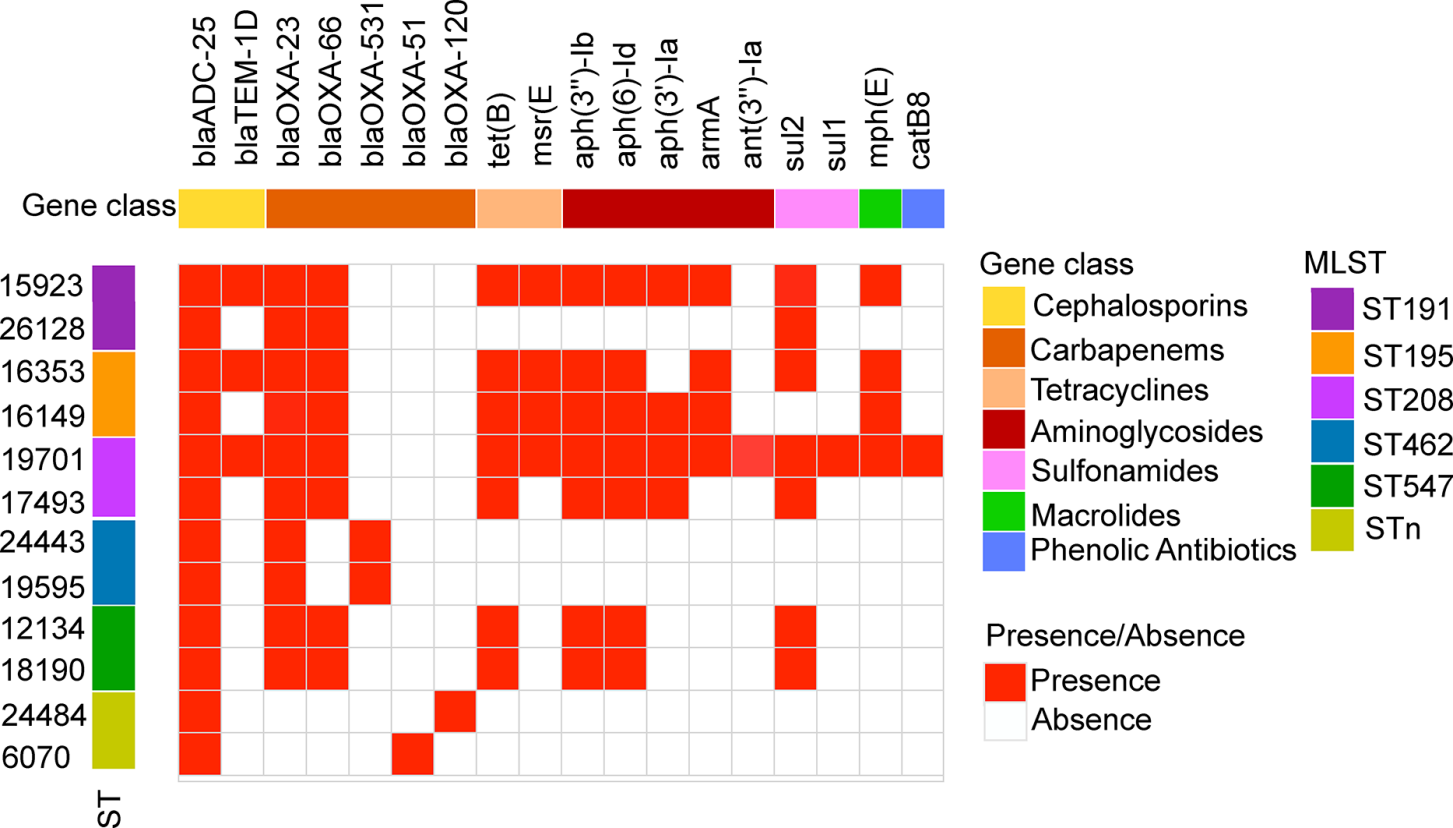
Antibiotic resistance gene profiles of *A. baumannii* DST and NST strains. The first row represents the resistance gene designation, and the second row represents the gene functional classification. The columns from left to right represent the strain name and ST type. Both identity and coverage are greater than 80% as the threshold for the presence of genes.

To explore why the DST group strains had higher antibiotic resistance rates than those of the NST group, the profile of resistance genes in each strain was analyzed using the ResFinder database. Although strains in the two groups had several resistance genes in common, including genes encoding aminoglycoside, β-lactam, and sulfonamide resistance, many other resistance genes were found only in the DST group, correlating with the differences in drug susceptibility observed for the two groups. These unique genes included *ant(3”)-Ia_1* and *armA_1* (aminoglycoside resistance), *bla*TEM-1D_1 (β-lactam resistance), *mph(E)_1* (macrolide resistance), and *sul1_5* (sulfonamide resistance; **Figure 3**). Interestingly, the only resistance genes unique to the NST group were β-lactam-related genes, such as *bla*OXA-120_1, *bla*OXA-51_1, and *bla*OXA-531_1 (**Figure 3**).

### 3.4 Transcriptome analysis

**Figure 4:**
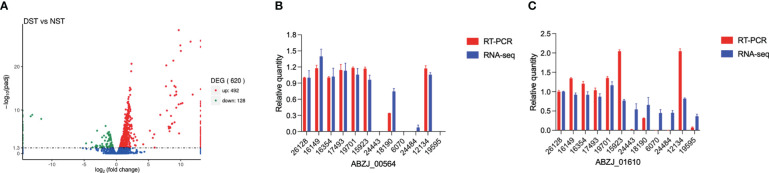
Relative expression of genes in DST and NST strains. **(A)** The y-axis shows -log_10_padj values for each gene, while the x-axis shows log_2_ fold change of DST vs NST. Red represents upregulation genes, and green represents downregulation genes; FDR ≤ 0.05 was the criterion for significant differences. **(B)**, **(C)** RNA-seq data of several highly expressed genes were validated using Q-PCR.

Transcriptome analysis showed that a total of 620 protein-coding genes were differentially expressed between the two groups (padj < 0.05), with 492 genes upregulated and 128 genes downregulated, in DST compared with NST, accounting for approximately 15% of all protein-coding genes (**Figure 4**). After screening by log_2_ FoldChange (DST/NST) ≥ 2, 128 genes were identified as being significantly upregulated in the DST group, including nine CPS synthesis-related genes, one efflux pump gene, and three T6SS secretion system-related genes. Loci corresponding to gene_ids for all these genes were identified in the genomes of the DST strains being analyzed (**Table S5**). After screening by log_2_ FoldChange (DST/NST) ≤-2, 53 genes were identified as being significantly downregulated in the DST group, including three genes related to CPS biosynthesis and six genes related to iron uptake (**Table S6**). Meanwhile, we randomly selected two genes for Q-PCR validation, respectively. The results showed that the relative trend in the expression of these genes, in strains from the different groups, was consistent with the transcriptomic data (**Figures 4B, C**).

**Figure 5:**
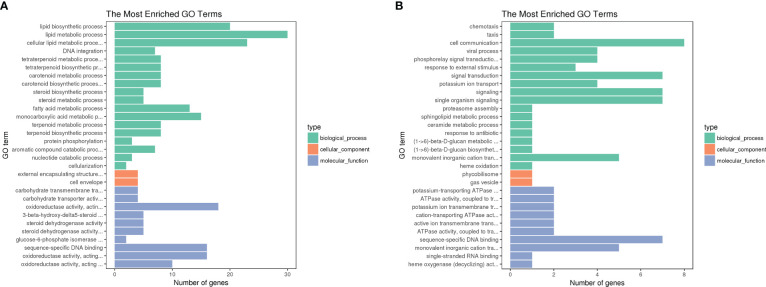
GO term functional analysis. Vertical coordinates are GO term functional modules; horizontal coordinates represent the number of enriched genes, where green represents biological processes, red represents cellular components, and gray represents molecular functions. We selected the 30 most significantly enriched GO terms to be shown in the figure, or all of them if there are less than 30. **(A)** GO enrichment of up-regulated DEGs in DST strains; **(B)** GO enrichment of down-regulated DEGs in DST strains. **(B)** Enrichment of genes downregulated in DST strains.

GO analysis showed that the most dramatically enriched gene set related to “lipid biosynthesis process,” which contains the *wecB*, *capD*, and *galE* genes involved in CPS synthesis, as well as the *fabB*, *fabF*, and *psd* genes involved in lipid syntheses. Other functional groups enriched in the DST upregulated genes group included “lipid metabolism process” and “carbohydrate transmembrane transport activity” (**Figure 5A**). The most significantly enriched, downregulated genes in the DST group were “potassium-transporting ATPase activity” and “ATPase activity, coupled to transmembrane movement of ions, phosphorylative mechanism,” with both of these functional sets containing the potassium ion transport genes *kdpA* and *kdpC*. Other functional sets with higher levels of enrichment in the DST downregulated genes group included “chemotaxis” and “taxis,” both of which include the *chpA* and *pilI* genes (**Figure 5B**).

**Figure 6:**
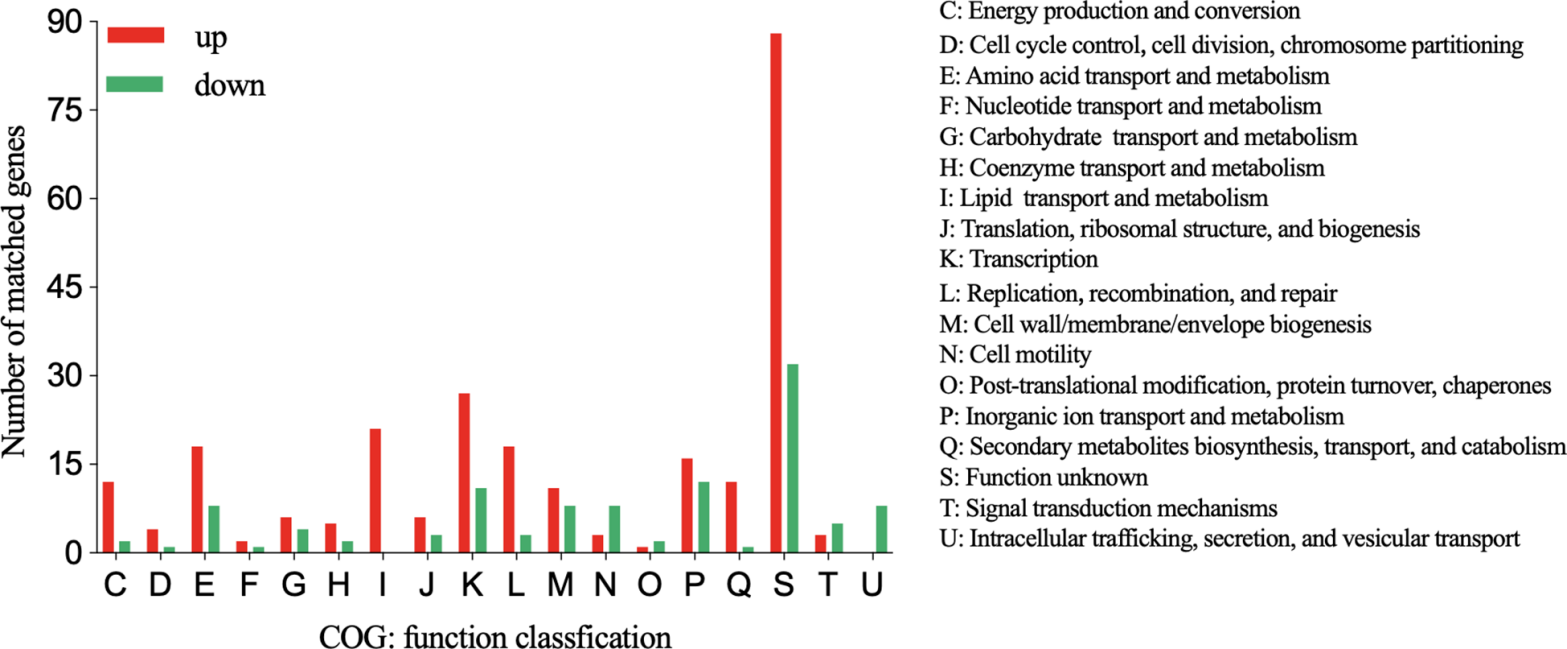
COG functional classification of up- and downregulated genes in the DST group relative to the NST group. Y-axis represents the number of up- or downregulated genes, and X-axis represents the COG functional classification.

Similar to the GO analysis, the COG analysis revealed that the “lipid transport and metabolism” category was enriched in the upregulated genes in the DST group (**Figure 6**). Additionally, the functional categories with the next largest differences in the number of genes expressed between the two groups were “Transcription” and “Replication,”. Although upregulated genes outnumbered downregulated genes in many functional categories, the “intracellular trafficking, secretion, and vesicular transport” category only contained downregulated genes. This suggests that strong secretion and vesicular transport abilities may be less important to DST strains.

**Figure 7:**
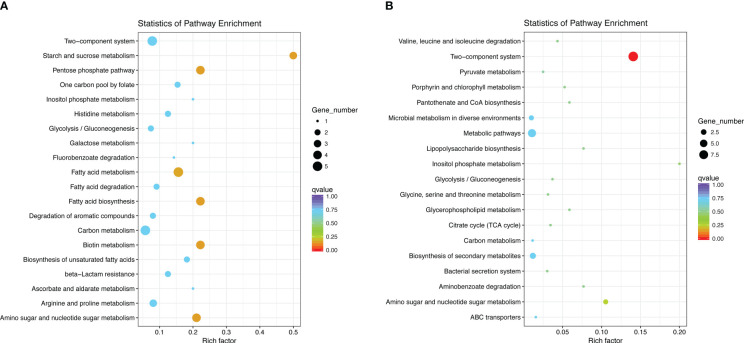
Statistics of pathway enrichment. We selected the 20 most significantly enriched pathway entries and displayed them in the graph. If the enriched pathway entries were less than 20, we displayed all of them. The ordinate represents the name of the pathway, the abscissa represents the Rich factor, the size of the point represents the number of DEGs in this pathway, and the color of the point corresponds to a different Q value. **(A)** KEGG enrichment of upregulated DEGs in DST strains; **(B)** KEGG enrichment of downregulated DEGs in DST strains.

The KEGG pathway enrichment analysis indicated that the most significant pathway enriched for upregulated genes was “starch and sucrose metabolism” (p = 0.0054), which contains three genes, *galU*, *ugd*, and *pgi*, related to the CPS biosynthesis. This is in agreement with the fact that the most significant terms in the GO analysis also contain three CPS synthesis-related genes. Other enriched pathways were “Pentose phosphate pathway,” “Biotin metabolism,” and “Fatty acid biosynthesis” (**Figure 7**). The most significant of the downregulated gene-enriched pathways were the “two-component systems” (p=1.20E-07), which include proteins associated with potassium uptake (kdpA, kdpB, and kdpC) and motility (pilJ, pilH, pilI, pilG, PilR).

### 3.5 Virulence and resistance genes in DEGs based on transcriptomic analysis

Differences in DEGs associated with virulence and antibiotic resistance were next investigated since these two phenotypes are closely linked to the clinical significance of bacteria and to their survival in the environment.

The VFDB database and the one-way blast approach for SwissProt annotation were combined to identify 42 virulence-related genes among the upregulated genes in strains of the DST group. The genes identified were similar to those identified by genomic analysis, the functions of which related mainly to CPS synthesis and transport (*wza*/*wzb*/*wzc*, etc.), iron uptake (Heme utilization genes, TonB-dependent iron carrier receptors, and iron regulatory proteins), type VI secretion systems (*vgrg*, *vgrgA*, contractile small subunits, and PAAR domain-containing proteins), and biofilm formation (*pilin*/*pgaB*, *ompW*, *oprM*, *abaR*). In addition, the toxin-antitoxin *cdiA/cdiB*, RNA chaperone *hfq*, sensor protein *pilS*, and the redox-sensitive transcriptional activator *soxR* were also highly expressed in strains of the DST group (**Table S7**). The functions of 36 virulence genes identified as downregulated in the DST groups mainly involved type IV pilus synthesis, and included genes encoding pilus assembly proteins (*pilC, pilB, pilJ, pilG, pilH, pilI, pilT*) and the pilus regulatory protein gene (*PilR*). In addition, genes downregulated in the DST group contained genes related to iron uptake, CPS synthesis, potassium transport, chemotactic and competition functions, type II secretion system, and phospholipase synthesis (**Table S8**).

Alignment of DEGs with the antibiotic resistance database ResFinder identified that genes encoding resistance to several antibiotic classes, including aminoglycosides, tetracyclines, β-lactam, chloramphenicol, and cationic antimicrobial peptide, were upregulated in the DST group (**Table S9**), while chlorhexidine, chloramphenicol, and aminoglycoside resistance genes were downregulated in the DST group (**Table S10**).

## 4 Discussion

*A. baumannii* is a significant cause of pneumonia and bloodstream infection in critically ill patients, and its high rate of multidrug resistance poses a great challenge to clinical treatment. Consequently, research on *A. baumannii* has focused mainly on revealing its antibiotic resistance mechanism. However, as the understanding of this pathogen deepens, it has become clear that virulence mechanisms of *A. baumannii* also play a very important role in human infections and disease prognosis. Sequence typing has attracted the attention of public health clinicians because it helps to study the transmission, outbreak, and prevalence of *A. baumannii* strains. Although sporadic studies have investigated the *A. baumannii* characteristics that contribute to specific STs becoming DSTs (Ali et al., 2017; Zahra et al., 2018; de Azevedo et al., 2019), research in this area is still in its infancy. Thus, a more integrated approach, which explores the relationship between the virulence, antibiotic resistance, and ST of specific dominant strains, is required.

Based on previously published studies, we identified ST191, ST195, and ST208 as the DSTs and ST 547 and ST462 as the NSTs of *A. baumannii*, and selected around ten strains of each ST for experimental research in this study. As serum complement killing assay is an important indicator of virulence of A. baumannii, we selected two strains from each of the three DST with high complement killing resistance, and two strains from each of the ST462, ST547 plus two STn that were sensitive to complement killing, for subsequent genomic and transcriptomic analyses.

As we expected, the DST group was more tolerant to various adverse environments, including oxidation, desiccation, and complement-mediated killing. This also correlated with the demonstration that the DST group possessed more genes related to CPS biosynthesis, and that the expression of these genes was higher than related genes in the NST strains. This is in agreement with previous studies, in which CPS was shown to play a vital role in desiccation resistance and anticomplement killing in *Acinetobacter* (Ophir and Gutnick, 1994; Tipton et al., 2018). Norton et al. (2013) revealed that desiccation stress can induce resistance to antibiotics, corresponding to higher resistance rates in the *A. baumannii* DST group (Norton et al., 2013).

Previous studies have shown that *A. baumannii* can form robust biofilms on wounds and medical devices such as endotracheal tubes (Thompson et al., 2014; Greene et al., 2016), as well as increase the tolerance to various extracellular stress (Espinal et al., 2012; Greene et al., 2016). To our surprise, the biofilm-forming ability of the DST group was not as strong as that of the NST group. Moreover, the biofilm formation-related genes *adeFGH*, *bap*, *csu* fimbriae, *pga/b/c/d*, *abaR*, and *abaI* all existed in the genomes of both groups, and there was no significant difference in the transcription of these genes by either group. The evolution of ST dominant strains may partly be driven by their sustained exposure to nutrient-deficient environments, and as such, 24 h biofilm formation experiments in a nutrient-rich environment do not accurately mimic these conditions. Alternatively, other, as yet unidentified, genes of *A. baumannii* may be important in the ability bacteria of this organism to form biofilms.

The evolutionary process of bacteria, on the one hand, desires to be resistant to as many antibiotics as possible, and on the other hand to retain their ability to survive in unfavorable environments and infect hosts, however, no bacteria can be highly pathogenic, pan-resistant, permanently prevalent and existence in all (Imperi et al., 2011; Da Silva and Domingues, 2017). This may explain why the DST group, which has a higher drug resistance rate and is more resistant to complement killing, has a weaker biofilm formation ability than the NST group.

Among the highly expressed virulence factors genes in the DST group (log_2_Foldchange(DST/NST) ≥2) were CPS biosynthesis and T6SS component-associated genes, whereas the downregulated virulence factors genes (log_2_Foldchange(DST/NST) ≤-2) were iron uptake related. This may be due to the fact that the capsule promotes the survival of *A. baumannii* both in the hospital setting and in patients. T6SS also contributes to the dominance of *A. baumannii* owing to its competition with other bacteria (Repizo et al., 2015; Weber et al., 2016). However, although the iron acquisition system helps bacteria to acquire iron in hosts with iron-deficient environments, it does not necessarily play an important role in promoting dominant clone formation (Penwell et al., 2015; Runci et al., 2019).

Interestingly, many type 4 pili genes related to adherence and motility, including *pilR*, *pilB*, *pilC*, *pilJ*, *pilG*, *pilH*, *pilI*, and *pilT*, were downregulated in the DST group, which showed higher resistance to serum complement. By contrast, a previous study showed that type IV pili of *A. baumannii* were upregulated while growing in serum (Jacobs et al., 2012). There are several conceivable explanations for this apparent anomaly. First, the media used for the growth of *A. baumannii* differed between the two studies. In our study, transcriptome analysis was carried out following growth of the bacterium in LB broth, whereas in the other study, the organism was grown in the presence of serum. Second, the *A. baumannii* strains used were different. Third, since glycosylation of pili has been shown to contribute to the survival of *P. aeruginosa* in the pulmonary environment (Smedley et al., 2005), post-transcriptional modifications may also play important roles in *A. baumannii* phenotypes; such modifications were not investigated in this study.

The transposon-sequence (Tn-seq) technique was used to identify 50 genes required for survival, in human serum, of a strain of *A. baumannii* isolated from a case of osteomyelitis (Sanchez-Larrayoz et al., 2017). Several of the genes identified in that study, such as CPS-related genes *wza*, and *wzb*, correlate with those we found to be upregulated in the DST group.

Lipid biosynthesis plays an important role in the synthesis of phospholipids in bacteria, and asymmetric bacterial cell membranes are an important permeability barrier that contributes to bacterial resistance to many antibiotics (Zhang and Rock, 2008; Simpson and Trent, 2019). This is consistent with one of our most important findings, namely, that genes associated with lipid biosynthesis and transport pathways are enriched in those genes upregulated in the DST group. Moreover, the upregulated *fabB* and *fabF* involved in this pathway exhibit a prolonged effect on fatty acid synthesis, and the knockdown of *fabB* in *E. coli* leads to unsaturated fatty acid nutrient deficiency and failure to grow properly (Wang and Cronan, 2004). The knockdown of *fabF* also reduces motility in *P. aeruginosa* (Overhage et al., 2007). To date, however, there is little information on the roles in the virulence and adaptation of *Acinetobacter baumannii* of many of the genes in this pathway.

Naturally, our study has some shortcomings; for example, the number of the strains used in the genome and transcriptome analysis is small and may not be particularly representative. What’s more, it would be impossible to collect and analyze all possible environmental conditions (e.g., temperature, humidity, rainfall, and other climatic factors) that may influence the evolution of the strains.

In conclusion, tolerance to desiccation, oxidation, and complement killing all play an important role in the evolution of DST *A. baumannii* strains, while CPS biosynthesis, T6SS, and especially lipid synthesis pathway-related genes also play an indispensable role in this process. Our study further revealed the relationship between antibiotic resistance, virulence, and specific dominant ST of *A. baumannii*, laying a foundation for better prevention, control, and treatment of infections by this organism in the future.

## Data availability statement

The datasets presented in this study can be found in online repositories. The names of the repository/repositories and accession number(s) can be found below: https://www.ncbi.nlm.nih.gov/,PRJNA906176.

## Author contributions

XK completed most of the experiments and wrote the first draft of the manuscript. TC and PL contributed in toxicity testing of bacteria analysis. LG and YZ performed the bioinformatics analysis. YX contributed to conception and design of the study and revised the manuscript. All authors contributed to the article and approved the submitted version.
