# Supplementary material for: Phenotypic and genomic comparison of dominant and nondominant sequence-type of Acinetobacter baumannii isolated in China
Source: Front Cell Infect Microbiol. 2023 Feb 20;13:1118285. doi: 10.3389/fcimb.2023.1118285 (PMC9986592; doi:10.3389/fcimb.2023.1118285)
Supplement: Supplementary file 1 [file DataSheet_1.docx]

Supplementary Material

Article Title

Kong^*^, Tao Chen, Lihua Guo, Yanzi Zhou, Ping Lu, Yonghong Xiao

*** Correspondence:** Corresponding Author: xiaoyonghong@zju.edu.cn

## Supplementary Figures


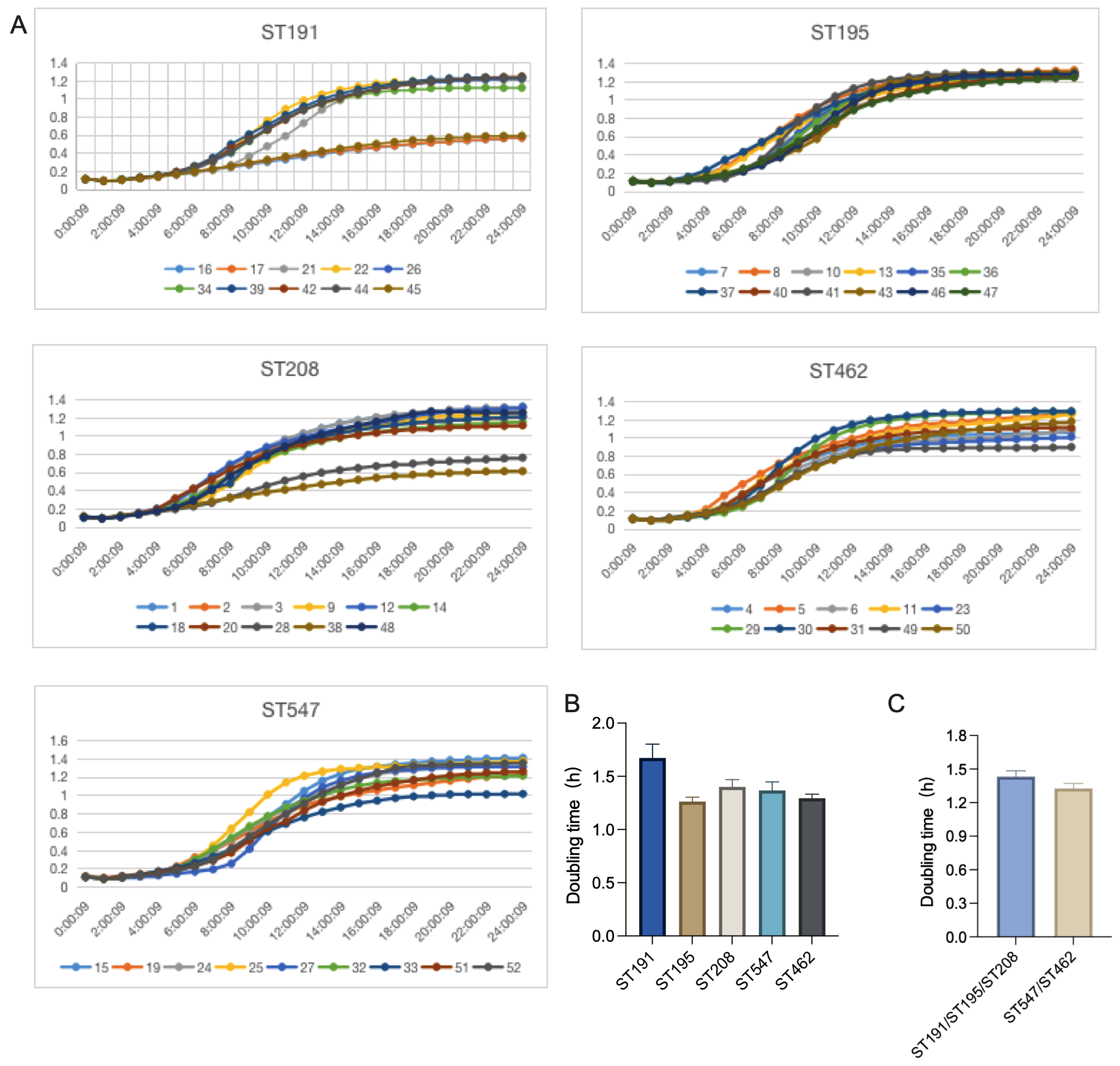


**Figure S1.** Growth curves of bacteria. A. Measurement of 24 h growth curves of each DST and NST strains used in the experiment. B. Doubling time of each ST type strains,. C.Doubling time between DST and NST strains.


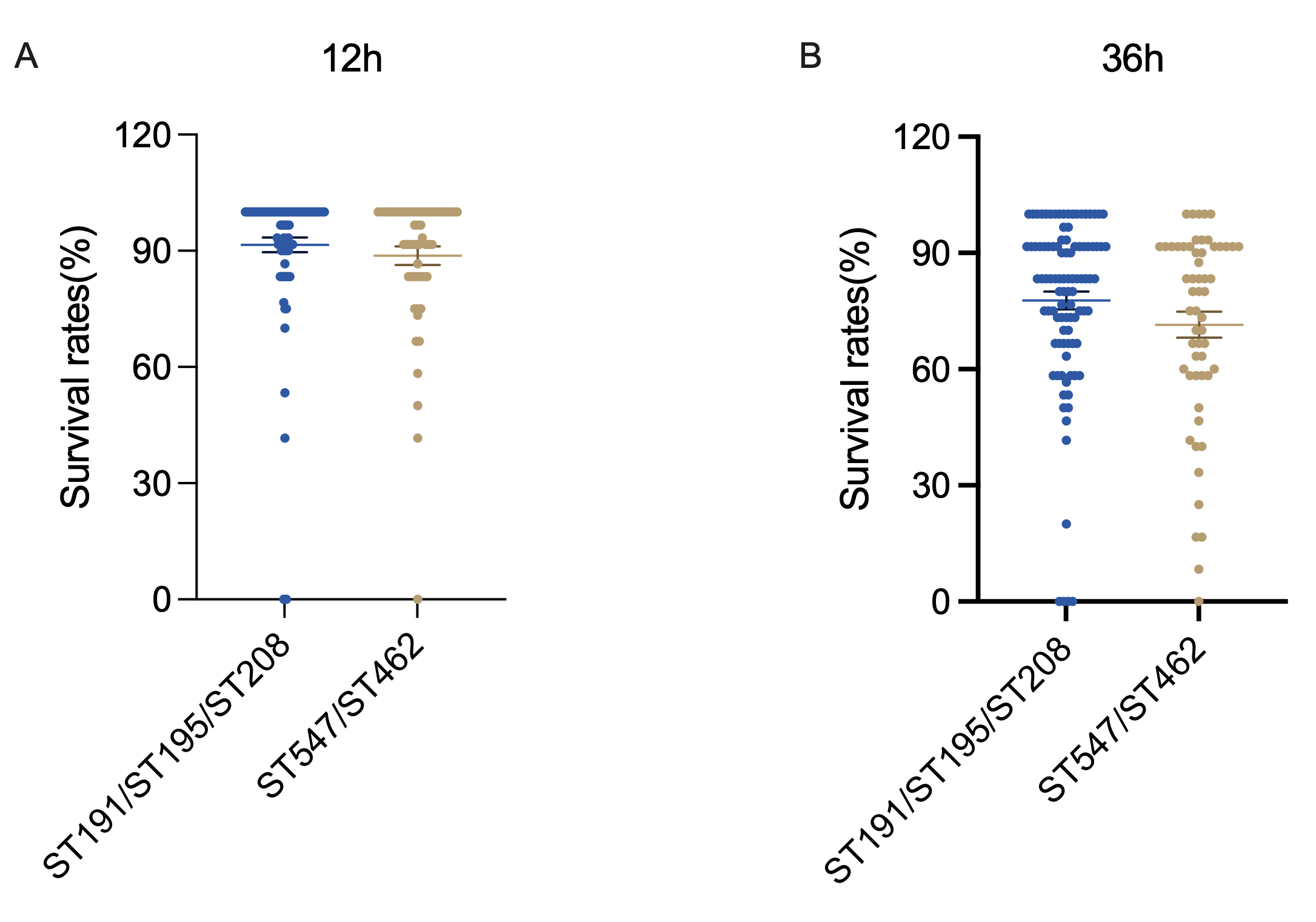


ns

ns

**Figure S2.** Bacterial infection of *G.mellonella.* A, Survival rate of *G.mellonella* in DST and NST groups after 12 h of infection. B, Survival rate of *G.mellonella* in DST and NST groups after 12 h of infection.


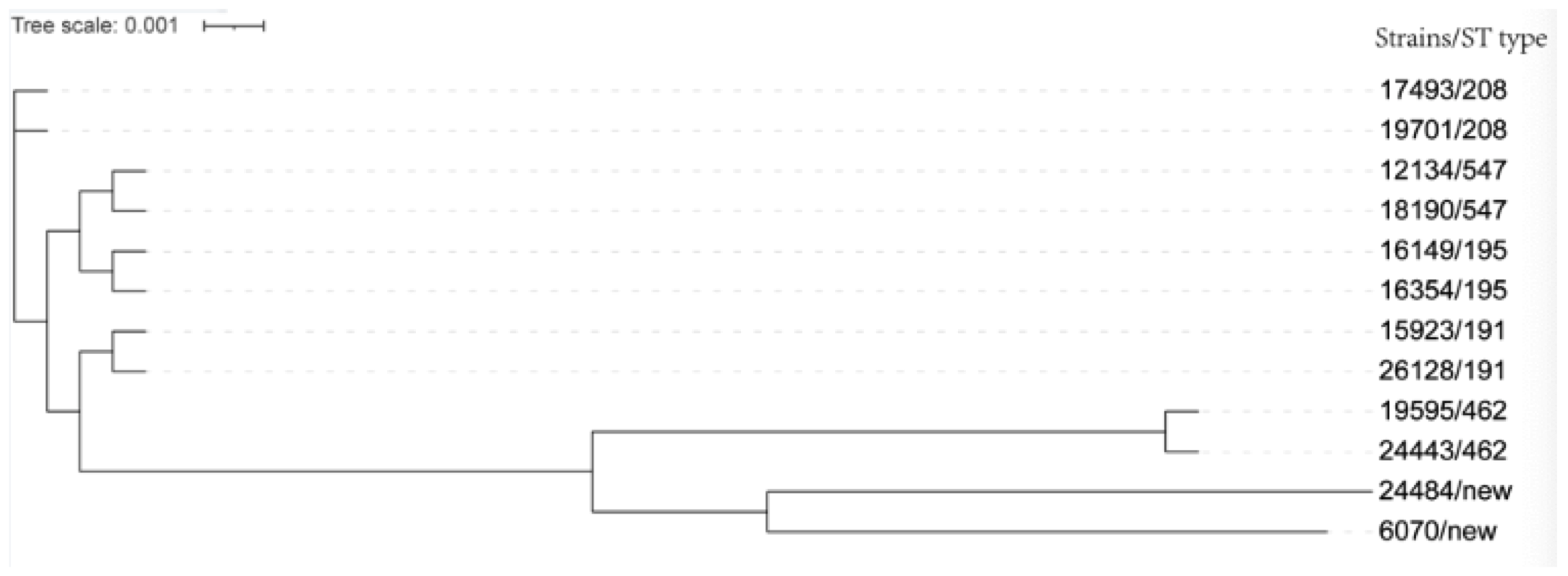


**Figure S3.** Phylogenetic tree among 12 strains

Table S1. **Strain Informations.** Color-marked strains are those used for genome and transcriptome analysis. Red indicates DST group and blue indicates NST group

| Numerbers | Sequence types | Samples | ID in the growth curve | |
| --- | --- | --- | --- | --- |
| SKLX11756 | ST191 | bl | 16 |  |
| SKLX12300 | ST191 | bl | 17 |  |
| SKLX13238 | ST191 | bl | 21 |  |
| SKLX13517 | ST191 | bl | 22 |  |
| SKLX16271 | ST191 | bl | 39 |  |
| SKLX18303 | ST191 | bl | 26 |  |
| SKLX25631 | ST191 | bl | 42 |  |
| SKLX4909 | ST191 | bl | 34 |  |
| SKLX15954 | ST195 | bl | 35 |  |
| SKLX15991 | ST195 | bl | 36 |  |
| SKLX16078 | ST195 | bl | 37 |  |
| SKLX16324 | ST195 | bl | 40 |  |
| SKLX24317 | ST195 | bl | 41 |  |
| SKLX27983 | ST195 | bl | 43 |  |
| SKLX6631 | ST195 | bl | 7 |  |
| SKLX6854 | ST195 | bl | 8 |  |
| SKLX8299 | ST195 | bl | 10 |  |
| SKLX9588 | ST195 | bl | 13 |  |
| SKLX10191 | ST208 | bl | 14 |  |
| SKLX12306 | ST208 | bl | 18 |  |
| SKLX13162 | ST208 | bl | 20 |  |
| SKLX16198 | ST208 | bl | 38 |  |
| SKLX237 | ST208 | bl | 1 |  |
| SKLX3833 | ST208 | bl | 3 |  |
| SKLX541 | ST208 | bl | 2 |  |
| SKLX7799 | ST208 | bl | 9 |  |
| SKLX9204 | ST208 | bl | 12 |  |
| SKLX13730 | ST462 | bl | 23 |  |
| SKLX4655 | ST462 | bl | 4 |  |
| SKLX4937 | ST462 | bl | 5 |  |
| SKLX5907 | ST462 | bl | 6 |  |
| SKLX78017 | ST462 | bl | 29 |  |
| SKLX80188 | ST462 | ur | 30 |  |
| SKLX8883 | ST462 | bl | 11 |  |
| SKLX98988 | ST462 | sp | 31 |  |
| SKLX11257 | ST547 | bl | 15 |  |
| SKLX112936 | ST547 | sp | 32 |  |
| SKLX112982 | ST547 | sp | 33 |  |
| SKLX12410 | ST547 | bl | 19 |  |
| SKLX14792 | ST547 | bl | 24 |  |
| SKLX18205 | ST547 | bl | 25 |  |
| SKLX18513 | ST547 | bl | 27 |  |
| SKLX15923 | ST191 | bl | 44 |  |
| SKLX26128 | ST191 | bl | 45 |  |
| SKLX16149 | ST195 | bl | 46 |  |
| SKLX16354 | ST195 | bl | 47 |  |
| SKLX17493 | ST208 | bl | 28 |  |
| SKLX19701 | ST208 | bl | 48 |  |
| SKLX19595 | ST462 | bl | 50 |  |
| SKLX24443 | ST462 | bl | 49 |  |
| SKLX12134 | ST547 | bl | 52 |  |
| SKLX18190 | ST547 | bl | 51 |  |
| SKLX24484 | STnew | bl |  |  |
| SKLX6070 | STnew | bl |  |  |

TableS2 Transcriptome sequencing quality control

| Sample name | Raw reads | Clean reads | clean bases | Error rate(%) | Q20(%) | Q30(%) | GC content(%) |
| --- | --- | --- | --- | --- | --- | --- | --- |
| A26128 | 19501236 | 19125642 | 2.87G | 0.02 | 98.41 | 94.74 | 41.26 |
| A26128_1 | 16817320 | 16511460 | 2.48G | 0.02 | 98.43 | 94.77 | 41.27 |
| A26128_2 | 19338336 | 19074862 | 2.86G | 0.02 | 98.52 | 94.96 | 41.28 |
| A16149 | 15911060 | 15635056 | 2.35G | 0.02 | 98.54 | 95.1 | 41.51 |
| A16149_1 | 21094568 | 20794212 | 3.12G | 0.02 | 98.5 | 95.02 | 42.06 |
| A16149_2 | 15959708 | 15537686 | 2.33G | 0.02 | 98.13 | 94.25 | 41.91 |
| A16354 | 16924196 | 16503886 | 2.48G | 0.02 | 98.62 | 95.24 | 41.75 |
| A16354_1 | 15452812 | 15227744 | 2.28G | 0.02 | 98.45 | 94.78 | 41.81 |
| A16354_2 | 18104824 | 17725652 | 2.66G | 0.02 | 98.18 | 94.32 | 39.69 |
| A17493 | 17260288 | 16854602 | 2.53G | 0.02 | 98.53 | 95.05 | 41.46 |
| A17493_1 | 19514690 | 19226690 | 2.88G | 0.02 | 98.49 | 94.97 | 41.76 |
| A17493_2 | 17780748 | 17359200 | 2.6G | 0.02 | 98.53 | 95.04 | 41.13 |
| A19701 | 14789200 | 14376232 | 2.16G | 0.02 | 98.45 | 94.91 | 41.25 |
| A19701_1 | 16774896 | 16306112 | 2.45G | 0.02 | 98.13 | 94.2 | 41.4 |
| A19701_2 | 17523084 | 17149784 | 2.57G | 0.02 | 98.6 | 95.19 | 41.39 |
| A15923 | 16713068 | 16292902 | 2.44G | 0.02 | 98.35 | 94.72 | 42.48 |
| A15923_1 | 21132956 | 20591720 | 3.09G | 0.02 | 98.61 | 95.25 | 42.26 |
| A15923_2 | 19369528 | 18730022 | 2.81G | 0.02 | 98.46 | 94.95 | 40.22 |
| B24443 | 18141680 | 17797104 | 2.67G | 0.02 | 98.2 | 94.2 | 41.37 |
| B24443_1 | 17140136 | 16567262 | 2.49G | 0.02 | 98.52 | 95.08 | 41.3 |
| B24443_2 | 17470808 | 17182050 | 2.58G | 0.02 | 98.65 | 95.33 | 41.59 |
| B18190 | 19250650 | 18891270 | 2.83G | 0.02 | 98.61 | 95.27 | 41.71 |
| B18190_1 | 17644028 | 17328266 | 2.6G | 0.02 | 98.38 | 94.67 | 41.2 |
| B18190_2 | 17127614 | 16769172 | 2.52G | 0.02 | 98.55 | 95.16 | 41.39 |
| B6070 | 16645616 | 16331234 | 2.45G | 0.02 | 98.53 | 95.04 | 41.57 |
| B6070_1 | 16114096 | 15736178 | 2.36G | 0.02 | 98.56 | 95.02 | 40.82 |
| B6070_2 | 15367158 | 15071890 | 2.26G | 0.02 | 98.6 | 95.22 | 39.74 |
| B24484 | 17394214 | 17036370 | 2.56G | 0.02 | 98.58 | 95.22 | 42.13 |
| B24484_1 | 18321692 | 18052514 | 2.71G | 0.02 | 98.43 | 94.79 | 41.25 |
| B24484_2 | 12703896 | 12344514 | 1.85G | 0.02 | 98.5 | 95.02 | 42.18 |
| B12134 | 16349118 | 16047468 | 2.41G | 0.02 | 98.65 | 95.35 | 41.98 |
| B12134_1 | 18365046 | 18135908 | 2.72G | 0.02 | 98.56 | 95.12 | 42.24 |
| B12134_2 | 16255416 | 15906100 | 2.39G | 0.02 | 98.22 | 94.45 | 40.55 |
| B19595 | 17691262 | 17353720 | 2.6G | 0.02 | 98.46 | 94.88 | 41.23 |
| B19595_1 | 20319234 | 19990498 | 3G | 0.02 | 98.51 | 95.04 | 41.23 |
| B19595_2 | 14296414 | 14058546 | 2.11G | 0.03 | 97.8 | 93.33 | 41.18 |

TableS3 Q-PCR for validating transcriptome results primer sequences

| Name | **Sequence (5'->3')** |
| --- | --- |
| ABZJ_00564 Forward primer | AGCATGGCGGATTCTATGGG |
| ABZJ_00564 Reverse primer | AATACGGCCACCTTTGCGTA |
| ABZJ_01610 Forward primer | CGGGCAATTAGACGTTGTGG |
| ABZJ_01610 Reverse primer | ACCACCATCTGGCACTGTTT |
| RecA_F | TACAGAAAGCTGGTGCATGG |
| RecA_R | TGCACCATTTGTGCCTGTAG |

Table S4 Genomic profile of strains and statistical analysis of contigs

| Strain | 15923 | 26128 | 16149 | 16354 | 17493 | 19701 | 12134 | 18190 | 19595 | 24443 | 24484 | 6070 |
| --- | --- | --- | --- | --- | --- | --- | --- | --- | --- | --- | --- | --- |
| SS | 4008614 | 3943960 | 3961167 | 3971098 | 3994932 | 4095330 | 3938982 | 3882765 | 4072210 | 4074076 | 3885146 | 3767945 |
| NOC | 63 | 65 | 51 | 54 | 58 | 47 | 71 | 52 | 39 | 30 | 121 | 151 |
| GC | 38.9 | 38.9 | 38.9 | 38.8 | 39 | 39 | 38.8 | 38.9 | 38.8 | 38.8 | 39 | 38.9 |
| SCS | 308 | 301 | 345 | 345 | 353 | 253 | 124 | 393 | 300 | 300 | 310 | 124 |
| MDS | 30368 | 27804 | 38150 | 43375 | 27736 | 51118 | 13877 | 38163 | 20726 | 30023 | 7097 | 5670 |
| MAS | 63628.8 | 60676.3 | 77669.9 | 73538.9 | 68878.1 | 87134.7 | 55478.6 | 74668.6 | 104415.6 | 135802.5 | 32108.6 | 24953.3 |
| LCS | 448572 | 355920 | 462285 | 462903 | 431319 | 431489 | 292899 | 343260 | 884066 | 1381510 | 340879 | 244108 |
| N50 | 169166 | 132440 | 207154 | 134156 | 168011 | 162885 | 164764 | 169250 | 347007 | 447367 | 97557 | 86126 |
| L50 | 8 | 10 | 7 | 9 | 8 | 8 | 10 | 8 | 4 | 3 | 12 | 14 |
| Note: SS: Sequence size, NOC: Number of contigs, GC: GC content (%), SCS: Shortest contig size, MDSS: Median sequence size, MAS: Mean sequence size, LCS: Longest contig size , | | | | | | | | | |  |  |  |

TableS5 The upregulated genes, after screening by log_2_ (DST/NST)≥2

| Gene_id | DST readcount | NST readcount | log_2_(DST/NST) | padj | product |
| --- | --- | --- | --- | --- | --- |
| ABZJ_00923 | 39.930 | 0.000 | Inf | 1.36E-11 | NA |
| ABZJ_00931 | 4.239 | 0.000 | Inf | 0.00017682 | NA |
| ABZJ_01002 | 67.812 | 0.000 | Inf | 0.0011416 | NA |
| ABZJ_01003 | 276.974 | 0.000 | Inf | 0.00020625 | NA |
| ABZJ_01004 | 53.919 | 0.000 | Inf | 0.0019677 | NA |
| ABZJ_01005 | 5.842 | 0.000 | Inf | 0.032402 | NA |
| ABZJ_01268 | 12.816 | 0.000 | Inf | 8.51E-07 | NA |
| ABZJ_01271 | 6.758 | 0.000 | Inf | 2.00E-05 | NA |
| ABZJ_01272 | 3.648 | 0.000 | Inf | 0.00079863 | hypothetical protein |
| ABZJ_01273 | 8.603 | 0.000 | Inf | 3.62E-06 | NA |
| ABZJ_01286 | 8.726 | 0.000 | Inf | 0.019241 | aminoglycoside N-acetyltransferase AAC(6')-Ib3 |
| ABZJ_01287 | 24.399 | 0.000 | Inf | 0.007756 | Aminoglycoside N(6&apos;)-acetyltransferase type 1 aacA4 |
| ABZJ_01288 | 893.341 | 0.000 | Inf | 4.58E-05 | Chloramphenicol acetyltransferase cat |
| ABZJ_02330 | 7.378 | 0.000 | Inf | 1.10E-07 | NA |
| ABZJ_02334 | 52.916 | 0.000 | Inf | 8.72E-12 | NA |
| ABZJ_02337 | 23.086 | 0.000 | Inf | 0.004129 | KilA-N domain-containing protein |
| ABZJ_02587 | 5.351 | 0.000 | Inf | 1.10E-05 | hypothetical protein |
| ABZJ_02590 | 111.245 | 0.000 | Inf | 2.97E-25 | NA |
| ABZJ_02593 | 13.175 | 0.000 | Inf | 1.07E-12 | NA |
| ABZJ_02599 | 31.011 | 0.000 | Inf | 2.87E-09 | NA |
| ABZJ_02603 | 7.175 | 0.000 | Inf | 6.62E-06 | NA |
| ABZJ_02604 | 1.990 | 0.000 | Inf | 0.038552 | NA |
| ABZJ_02605 | 14.235 | 0.000 | Inf | 5.97E-08 | NA |
| ABZJ_02606 | 5.322 | 0.000 | Inf | 1.39E-05 | NA |
| ABZJ_02607 | 3.953 | 0.000 | Inf | 0.00033369 | NA |
| ABZJ_02608 | 8.186 | 0.000 | Inf | 2.22E-06 | NA |
| ABZJ_02609 | 5.351 | 0.000 | Inf | 1.44E-05 | NA |
| ABZJ_02610 | 7.467 | 0.000 | Inf | 4.46E-05 | NA |
| ABZJ_02611 | 18.784 | 0.000 | Inf | 1.37E-07 | NA |
| ABZJ_02612 | 17.345 | 0.000 | Inf | 1.81E-09 | NA |
| ABZJ_02613 | 29.798 | 0.000 | Inf | 3.89E-08 | Uncharacterized protein |
| ABZJ_02615 | 20.037 | 0.000 | Inf | 3.08E-12 | Uncharacterized protein |
| ABZJ_02617 | 27.278 | 0.000 | Inf | 1.38E-10 | Uncharacterized protein |
| ABZJ_02632 | 3.987 | 0.000 | Inf | 0.0024503 | NA |
| ABZJ_02642 | 12.609 | 0.000 | Inf | 4.31E-08 | NA |
| ABZJ_02643 | 4.380 | 0.000 | Inf | 0.0001239 | NA |
| ABZJ_02645 | 8.793 | 0.000 | Inf | 9.75E-08 | Uncharacterized adenine-specific methylase |
| ABZJ_02646 | 2.110 | 0.000 | Inf | 0.028593 | NA |
| ABZJ_03344 | 80.132 | 0.000 | Inf | 0.00093403 | NA |
| ABZJ_03345 | 136.384 | 0.000 | Inf | 0.0004741 | NA |
| ABZJ_p00011 | 19.519 | 0.000 | Inf | 1.58E-15 | NA |
| ABZJ_p00012 | 135.694 | 0.000 | Inf | 1.15E-26 | Uncharacterized protein YcbJ |
| ABZJ_p00016 | 5.757 | 0.000 | Inf | 6.40E-06 | 16S rRNA (guanine(1405)-N(7))-methyltransferase |
| ABZJ_p00017 | 2.829 | 0.000 | Inf | 0.0064817 | Probable transposase for transposon Tn903 |
| EBG00001093351 | 9.009 | 0.000 | Inf | 0.02196 | NA |
| sRNA00010 | 3.701 | 0.000 | Inf | 0.00067349 | NA |
| sRNA00144 | 58.806 | 0.000 | Inf | 0.0014737 | NA |
| ABZJ_00924 | 198.064 | 0.053 | 11.86 | 2.79E-14 | Putative lambdoid prophage e14 repressor protein |
| ABZJ_02620 | 347.520 | 0.117 | 11.53 | 1.82E-26 | NA |
| ABZJ_02601 | 83.614 | 0.068 | 10.267 | 2.56E-12 | NA |
| ABZJ_p00004 | 3173.346 | 3.046 | 10.025 | 2.60E-24 | Aminoglycoside 3&apos;-phosphotransferase |
| ABZJ_02371 | 307.896 | 0.301 | 10 | 3.71E-12 | NA |
| ABZJ_02648 | 290.109 | 0.294 | 9.9479 | 1.35E-25 | site-specific integrase |
| Novel00005 | 432.231 | 0.502 | 9.7503 | 2.42E-05 | NA |
| ABZJ_02586 | 1460.616 | 1.760 | 9.6972 | 4.19E-29 | DUF4760 domain-containing protein |
| ABZJ_01269 | 111.267 | 0.136 | 9.6795 | 5.14E-12 | Uncharacterized protein |
| ABZJ_02614 | 199.122 | 0.312 | 9.3182 | 5.77E-15 | NA |
| ABZJ_02602 | 112.417 | 0.176 | 9.3171 | 2.79E-14 | NA |
| ABZJ_02335 | 57.889 | 0.091 | 9.3149 | 2.96E-11 | NA |
| ABZJ_02616 | 40.296 | 0.068 | 9.2142 | 5.32E-16 | Uncharacterized protein |
| ABZJ_01267 | 487.108 | 0.906 | 9.0711 | 4.57E-14 | Uncharacterized protein |
| ABZJ_00076 | 843.759 | 1.589 | 9.0527 | 6.68E-08 | polysaccharide biosynthesis/export family protein wza |
| ABZJ_02589 | 30.657 | 0.059 | 9.0275 | 2.93E-16 | NA |
| ABZJ_02633 | 164.496 | 0.352 | 8.8663 | 4.39E-22 | HTH-type transcriptional regulator for conjugative elemen |
| ABZJ_00092 | 1308.475 | 3.148 | 8.6991 | 1.29E-07 | Uncharacterized sugar transferase EpsL |
| ABZJ_00094 | 928.071 | 2.328 | 8.6393 | 1.56E-07 | UDP-glucose 6-dehydrogenase ugd |
| ABZJ_01261 | 34.910 | 0.091 | 8.5852 | 2.03E-17 | hypothetical protein |
| ABZJ_01270 | 45.650 | 0.127 | 8.4943 | 2.29E-09 | Uncharacterized protein |
| ABZJ_02596 | 19.728 | 0.059 | 8.3915 | 4.20E-14 | NA |
| ABZJ_00090 | 262.488 | 0.782 | 8.3911 | 1.24E-06 | Probable glycosyltransferase WbjE |
| ABZJ_00091 | 262.522 | 0.805 | 8.3493 | 1.27E-06 | UDP-glucose 4-epimerase galE |
| ABZJ_00088 | 792.116 | 2.446 | 8.3394 | 7.97E-07 | UDP-2-acetamido-2,6-beta-L-arabino-hexul-4-ose reductase wbjC |
| ABZJ_00075 | 38.880 | 0.127 | 8.2627 | 7.60E-06 | low molecular weight phosphotyrosine protein phosphatase wzb |
| ABZJ_00089 | 649.204 | 2.266 | 8.1624 | 1.79E-06 | UDP-N-acetylglucosamine 2-epimerase wecB |
| ABZJ_00087 | 1907.104 | 6.873 | 8.1163 | 6.62E-07 | UDP-glucose 4-epimerase capD |
| ABZJ_02600 | 118.161 | 0.511 | 7.8526 | 1.32E-10 | NA |
| ABZJ_02585 | 237.166 | 1.096 | 7.7581 | 5.49E-21 | hypothetical protein |
| ABZJ_02595 | 45.273 | 0.217 | 7.7016 | 3.54E-16 | NA |
| ABZJ_02591 | 10.341 | 0.091 | 6.8299 | 1.29E-09 | NA |
| ABZJ_03343 | 36.183 | 0.590 | 5.9381 | 0.04393 | NA |
| ABZJ_02647 | 8.018 | 0.136 | 5.8849 | 3.89E-05 | hypothetical protein |
| ABZJ_02644 | 4.614 | 0.091 | 5.6657 | 0.00025905 | hypothetical protein |
| ABZJ_02598 | 7.474 | 0.202 | 5.2113 | 5.83E-06 | NA |
| ABZJ_p00005 | 4.410 | 0.244 | 4.1757 | 0.0076553 | Transposase for insertion sequence-like element |
| ABZJ_02367 | 15.364 | 1.656 | 3.2138 | 0.00090078 | hypothetical protein |
| sRNA00018 | 11.102 | 1.373 | 3.0153 | 0.014809 | NA |
| ABZJ_p00006 | 10.925 | 1.399 | 2.9648 | 0.00014976 | replication initiation protein |
| ABZJ_02639 | 28.593 | 3.885 | 2.8798 | 0.011803 | hypothetical protein |
| ABZJ_01695 | 283.121 | 41.228 | 2.7797 | 3.92E-05 | hypothetical protein |
| ABZJ_00722 | 9.301 | 1.466 | 2.6657 | 0.0012777 | NA |
| ABZJ_01297 | 21.815 | 3.541 | 2.6229 | 0.00034224 | SDR family NAD(P)-dependent oxidoreductase |
| sRNA00083 | 25.880 | 4.816 | 2.4261 | 0.00040518 | NA |
| ABZJ_00904 | 186.251 | 35.008 | 2.4115 | 8.60E-09 | NA |
| Novel00042 | 386.300 | 73.384 | 2.3962 | 1.94E-06 | NA |
| Novel00039 | 948.862 | 184.735 | 2.3607 | 6.48E-07 | NA |
| ABZJ_02014 | 807.994 | 158.495 | 2.3499 | 2.30E-21 | PAAR domain-containing protein |
| Novel00041 | 539.348 | 106.377 | 2.342 | 2.09E-06 | NA |
| Novel00040 | 865.030 | 171.944 | 2.3308 | 3.82E-07 | NA |
| ABZJ_00906 | 83.689 | 16.750 | 2.3209 | 2.36E-07 | NA |
| ABZJ_02015 | 929.011 | 188.177 | 2.3036 | 1.22E-19 | hypothetical protein |
| ABZJ_01265 | 3483.511 | 714.994 | 2.2845 | 0.00021955 | type VI secretion system tip protein VgrG |
| ABZJ_02622 | 21.339 | 4.408 | 2.2753 | 1.96E-05 | NA |
| ABZJ_02044 | 369.244 | 78.616 | 2.2317 | 1.73E-15 | NA |
| Novel00037 | 1556.476 | 339.341 | 2.1975 | 1.65E-08 | NA |
| ABZJ_tRNA52 | 66.916 | 14.694 | 2.1871 | 0.02515 | tRNA-Leu |
| ABZJ_02031 | 225.997 | 50.291 | 2.1679 | 1.73E-10 | LuxR family transcriptional regulator |
| ABZJ_02121 | 91.860 | 20.461 | 2.1666 | 6.70E-11 | NA |
| ABZJ_00905 | 806.169 | 180.390 | 2.16 | 3.22E-07 | NA |
| ABZJ_02096 | 76.702 | 17.190 | 2.1577 | 4.94E-09 | hypothetical protein |
| ABZJ_01419 | 2405.716 | 541.420 | 2.1516 | 3.29E-14 | RHS domain-containing protein |
| ABZJ_00911 | 19.629 | 4.532 | 2.1146 | 8.51E-05 | NA |
| ABZJ_03753 | 710.233 | 165.382 | 2.1025 | 7.56E-16 | NA |
| ABZJ_02019 | 29.761 | 6.960 | 2.0962 | 0.0039156 | TetR/AcrR family transcriptional regulator |
| ABZJ_00907 | 368.287 | 86.348 | 2.0926 | 6.17E-07 | NA |
| ABZJ_02795 | 31.944 | 7.558 | 2.0795 | 3.10E-06 | type II toxin-antitoxin system Phd/YefM family antitoxin |
| ABZJ_02118 | 147.809 | 35.084 | 2.0749 | 1.93E-11 | hypothetical protein |
| ABZJ_02059 | 76.330 | 18.120 | 2.0747 | 2.68E-09 | NA |
| ABZJ_01420 | 532.778 | 126.889 | 2.07 | 3.18E-15 | hypothetical protein |
| ABZJ_01443 | 647.313 | 154.728 | 2.0647 | 2.96E-15 | NA |
| ABZJ_01173 | 445.645 | 106.634 | 2.0632 | 4.02E-11 | NA |
| ABZJ_02791 | 6.910 | 1.681 | 2.0398 | 0.0472 | HTH-type transcriptional regulator LmrA |
| ABZJ_03260 | 22.402 | 5.454 | 2.0381 | 0.00011459 | NA |
| ABZJ_tRNA29 | 19.332 | 4.729 | 2.0312 | 0.012327 | NA |
| ABZJ_00727 | 903.811 | 221.779 | 2.0269 | 0.0080947 | NA |
| Novel00038 | 1090.009 | 267.893 | 2.0246 | 3.68E-08 | NA |
| ABZJ_02129 | 12.592 | 3.128 | 2.0093 | 0.0051272 | NA |
| ABZJ_02095 | 578.337 | 144.278 | 2.0031 | 2.35E-11 | amidohydrolase |
| ABZJ_01406 | 497.065 | 124.137 | 2.0015 | 1.24E-11 | NA |

Note: #Inf means the readcounts value of the NST group is 0; #NAME? means the readcounts value of the NST group is 0. padj: p-value after multiple hypothesis testing correction. NA means No annotation to the product for this gene

Table S6 The downregulated genes, after screening by log_2_ (DST/NST)≤-2

| Gene_id | DST readcount | NST readcount | log_2_(DST/NST) | padj | product |
| --- | --- | --- | --- | --- | --- |
| ABZJ_00080 | 0.046 | 459.587 | -13.285 | 2.81E-09 | putative UDP-N-acetylglucosamine 2-epimerase |
| ABZJ_00081 | 0.092 | 776.404 | -13.041 | 1.27E-09 | N-acetylneuraminate synthase |
| ABZJ_00078 | 0.092 | 295.752 | -11.649 | 1.09E-08 | UDP-N-acetylglucosamine 4,6-dehydratase |
| ABZJ_01015 | 36.227 | 942.693 | -4.7016 | 0.0047382 | hypothetical protein CV956_014710 |
| ABZJ_01014 | 65.963 | 940.506 | -3.8337 | 0.0026886 | outer membrane receptor protein |
| ABZJ_02322 | 33.257 | 428.768 | -3.6885 | 0.037896 | transposase family protein |
| ABZJ_01016 | 11.704 | 111.595 | -3.2533 | 0.026481 | peptide signal protein |
| ABZJ_01012 | 21.826 | 191.379 | -3.1323 | 0.013259 | RNA polymerase subunit sigma |
| Novel00058 | 67.905 | 584.404 | -3.1054 | 0.033564 | IS256 family transposase ISAba26 |
| ABZJ_00229 | 29.218 | 232.816 | -2.9943 | 5.71E-06 | ATPase |
| ABZJ_01017 | 3.265 | 25.092 | -2.9419 | 0.018 | energy transducer TonB |
| ABZJ_02224 | 154.264 | 885.851 | -2.5217 | 0.014904 | DUF2057 domain-containing protein |
| ABZJ_01018 | 25.753 | 147.223 | -2.5152 | 0.043068 | heme oxygenase family protein |
| ABZJ_01019 | 9.340 | 48.315 | -2.371 | 0.033718 | hypothetical protein AQ974_08620 |
| ABZJ_01186 | 4.439 | 21.871 | -2.3008 | 0.040189 | phage family protein |
| ABZJ_01013 | 18.308 | 85.798 | -2.2285 | 0.049113 | iron ABC transporter permease |
| ABZJ_01641 | 102.389 | 471.224 | -2.2024 | 0.00019722 | hypothetical protein HMPREF0022_03908 |
| ABZJ_01594 | 41.858 | 191.823 | -2.1962 | 0.0062591 | allantoin permease |
| ABZJ_03605 | 2.442 | 11.157 | -2.1919 | 0.019507 | hypothetical protein A7L05_04600 |
| ABZJ_02056 | 63.955 | 276.434 | -2.1118 | 0.020336 | ABC transporter substrate-binding protein |
| ABZJ_02853 | 0.000 | 651.182 | #NAME? | 1.32E-10 | hypothetical protein A7A68_15210 |
| ABZJ_00082 | 0.000 | 352.140 | #NAME? | 1.43E-09 | alcohol dehydrogenase |
| ABZJ_00079 | 0.000 | 345.546 | #NAME? | 1.70E-09 | aminotransferase DegT |
| ABZJ_02845 | 0.000 | 236.808 | #NAME? | 3.51E-09 | hypothetical protein |
| ABZJ_02846 | 0.000 | 218.786 | #NAME? | 4.94E-08 | hypothetical protein |
| ABZJ_02866 | 0.000 | 56.148 | #NAME? | 1.92E-07 | phage tail protein |
| ABZJ_02870 | 0.000 | 49.590 | #NAME? | 2.69E-07 | hypothetical protein CEJ57_04460 |
| ABZJ_02825 | 0.000 | 35.651 | #NAME? | 4.68E-07 | transcriptional regulator |
| ABZJ_02865 | 0.000 | 34.490 | #NAME? | 9.76E-07 | hypothetical protein CEJ80_00770 |
| ABZJ_02856 | 0.000 | 29.986 | #NAME? | 1.32E-06 | phage head morphogenesis protein |
| ABZJ_02852 | 0.000 | 32.186 | #NAME? | 1.33E-06 | hypothetical protein CEJ66_00335 |
| ABZJ_02356 | 0.000 | 9.092 | #NAME? | 2.81E-06 | methyltransferase |
| ABZJ_02855 | 0.000 | 24.320 | #NAME? | 4.19E-06 | DUF935 domain-containing protein |
| ABZJ_02363 | 0.000 | 5.590 | #NAME? | 4.96E-06 | hypothetical protein ASV48_18995 |
| ABZJ_02864 | 0.000 | 21.596 | #NAME? | 5.51E-06 | hypothetical protein AZE33_12470 |
| ABZJ_02854 | 0.000 | 10.243 | #NAME? | 4.57E-05 | restriction endonuclease subunit M |
| ABZJ_02868 | 0.000 | 7.989 | #NAME? | 6.01E-05 | hypothetical protein A7A68_15285 |
| ABZJ_02857 | 0.000 | 8.660 | #NAME? | 7.70E-05 | virion morphogenesis protein |
| ABZJ_02858 | 0.000 | 7.840 | #NAME? | 7.70E-05 | hypothetical protein CEJ79_11920 |
| ABZJ_02877 | 0.000 | 8.148 | #NAME? | 0.00018773 | hypothetical protein AZE33_12535 |
| ABZJ_02839 | 0.000 | 5.208 | #NAME? | 0.000229 | hypothetical protein ABR2091_2568 |
| ABZJ_02875 | 0.000 | 5.763 | #NAME? | 0.00029114 | hypothetical protein CEJ74_03010 |
| ABZJ_02860 | 0.000 | 4.942 | #NAME? | 0.00032497 | putative major head subunit protein |
| ABZJ_02840 | 0.000 | 4.778 | #NAME? | 0.00034852 | transposase |
| ABZJ_02871 | 0.000 | 4.062 | #NAME? | 0.0007838 | multidrug DMT transporter |
| ABZJ_02826 | 0.000 | 4.184 | #NAME? | 0.00082452 | GemA protein |
| ABZJ_02829 | 0.000 | 3.441 | #NAME? | 0.000921 | hypothetical protein APC61_16110 |
| ABZJ_02862 | 0.000 | 3.005 | #NAME? | 0.0028164 | hypothetical protein CEJ66_00385 |
| ABZJ_02834 | 0.000 | 2.634 | #NAME? | 0.0045069 | hypothetical protein AZE33_12330 |
| ABZJ_00083 | 0.000 | 2.519 | #NAME? | 0.0060767 | CMP-N-acetlyneuraminic acid synthetase |
| ABZJ_02835 | 0.000 | 2.080 | #NAME? | 0.021456 | hypothetical protein CEJ65_08115 |
| ABZJ_02847 | 0.000 | 1.802 | #NAME? | 0.03823 | hypothetical protein CKN52_04105 |
| ABZJ_02841 | 0.000 | 1.701 | #NAME? | 0.04933 | hypothetical protein CEJ78_12915 |

Table S7 Virulence genes upregulated in DST group

| Category | geneID | log_2_（DST/NST） | padj | Product |
| --- | --- | --- | --- | --- |
| Capsule | ABZJ_00076 | 9.05 | 6.68E-08 | polysaccharide biosynthesis/export family protein,Wza |
| Capsule | ABZJ_00092 | 8.7 | 1.29E-07 | Uncharacterized sugartransferase,EpsL |
| Capsule | ABZJ_00094 | 8.64 | 1.56E-07 | UDP-glucose 6-dehydrogenase |
| Capsule | ABZJ_00090 | 8.39 | 1.24E-06 | Probable glycosyltransferase,WbjE |
| Capsule | ABZJ_00091 | 8.35 | 1.27E-06 | UDP-glucose 4-epimerase,GalE |
| Capsule | ABZJ_00088 | 8.34 | 7.97E-07 | UDP-2-acetamido-2,6-beta-L-arabino-hexul-4-ose reductase,wbjC |
| Capsule | ABZJ_00075 | 8.26 | 7.60E-06 | Low molecular weight protein-tyrosine-phosphatase wzb |
| Capsule | ABZJ_00089 | 8.16 | 1.79E-06 | UDP-N-acetylglucosamine 2-epimerase,WecB |
| Capsule | ABZJ_00087 | 8.12 | 6.62E-07 | UDP-glucose 4-epimerase |
| Capsule | ABZJ_00093 | 1.52 | 1.94E-04 | UTP--glucose-1-phosphate uridylyltransferase,GalU |
| Capsule | ABZJ_00095 | 0.98 | 8.91E-03 | Glucose-6-phosphate isomerase,pgi |
| Iron uptake | ABZJ_02072 | 1.64 | 2.57E-08 | Hemolysin-type calcium-binding region |
| Iron uptake | ABZJ_01858 | 1.62 | 1.22E-09 | Putative TonB-dependent receptor |
| Iron uptake | ABZJ_03308 | 1.54 | 1.09E-08 | Outer membrane vitamin B12 receptor BtuB |
| Iron uptake | ABZJ_01795 | 1.43 | 7.26E-09 | TonB-dependent receptor |
| Iron uptake | ABZJ_01216 | 0.68 | 4.34E-02 | TonB-dependent receptor |
| Iron uptake | ABZJ_00789 | 0.57 | 2.26E-02 | outer membrane autotransporter barrel domain |
| T6SS | ABZJ_02014 | 2.35 | 2.30E-21 | PAAR domain-containing protein |
| T6SS | ABZJ_01265 | 2.28 | 2.20E-04 | type VI secretion system tip protein VgrG |
| T6SS | ABZJ_01418 | 1.33 | 3.93E-08 | type VI secretion system tip protein VgrG |
| T6SS | ABZJ_03754 | 0.93 | 2.53E-04 | type VI secretion system tip protein VgrG |
| T6SS | ABZJ_01454 | 1.24 | 5.89E-05 | type VI secretion system contractile sheath small subunit |
| biofilm formation | ABZJ_03565 | 1.75 | 6.61E-09 | pilin |
| biofilm formation | ABZJ_01980 | 1.33 | 9.97E-06 | type 1 fimbrial protein |
| biofilm formation | ABZJ_02515 | 0.55 | 4.59E-02 | poly-beta-1%2C6-N-acetyl-D-glucosamine N-deacetylase PgaB |
| outer membrane protein | ABZJ_02720 | 1.9 | 3.54E-12 | OmpW family protein |
| outer membrane protein | ABZJ_02003 | 1.11 | 1.34E-02 | OprM protein |
| Toxin-antitoxin | ABZJ_02795 | 2.08 | 3.10E-06 | type II toxin-antitoxin system Phd/YefM family antitoxin |
| Toxin-antitoxin | ABZJ_02343 | 1.85 | 3.39E-02 | type II toxin-antitoxin system HicB family antitoxin |
| Toxin-antitoxin | ABZJ_02091 | 1.46 | 2.40E-05 | Toxin CdiA |
| Toxin-antitoxin | ABZJ_02092 | 1.34 | 5.86E-03 | Toxin CdiB |
| Toxin-antitoxin | ABZJ_00243 | 1.25 | 1.24E-02 | zeta toxin family protein |
| Toxin-antitoxin | ABZJ_00975 | 1.11 | 4.06E-03 | RelE-like toxin protein |
| Quorum sensing | ABZJ_00140 | 0.95 | 4.74E-03 | LuxR family transcriptional regulator abaR |
| others | ABZJ_02645 | Inf | 9.75E-08 | adenine-specific methylase |
| others | ABZJ_p00016 | Inf | 6.40E-06 | 16S rRNA (guanine(1405)-N(7))-methyltransferase |
| others | ABZJ_02116 | 1.72 | 1.34E-08 | Catalase katA |
| others | ABZJ_02097 | 1.67 | 1.49E-07 | Glutathione-regulated potassium-efflux system protein |
| others | ABZJ_00740 | 1.4 | 5.13E-03 | Sensor protein PilS |
| others | ABZJ_01482 | 1.01 | 1.22E-03 | redox-sensitive transcriptional activator SoxR |
| others | ABZJ_02465 | 0.66 | 1.22E-02 | RNA chaperone Hfq |
| others | ABZJ_03770 | 0.59 | 3.84E-02 | phosphatidylserine decarboxylase |

Table S8 Virulence genes downregulated in DST group

| Category | gene_id | log_2_（DST/NST） | padj | products |
| --- | --- | --- | --- | --- |
| Pili | ABZJ_00373 | -1.92 | 5.24E-06 | type IV-A pilus assembly ATPase PilB |
| Pili | ABZJ_03243 | -1.24 | 7.02E-07 | type IV pilus biogenesis protein PilJ |
| Pili | ABZJ_03246 | -1.15 | 1.02E-04 | Protein PilG |
| Pili | ABZJ_03244 | -1.15 | 5.91E-05 | chemotaxis protein CheW/pill |
| Pili | ABZJ_00372 | -1.15 | 4.72E-06 | Type 4 fimbrial assembly protein PilC |
| Pili | ABZJ_00887 | -1.02 | 2.80E-05 | type IV pili twitching motility protein PilT |
| Pili | ABZJ_03576 | -0.9 | 1.03E-03 | Type IV pilus biogenesis protein PilP |
| Pili | ABZJ_00284 | -0.86 | 1.71E-03 | Type 4 fimbriae expression regulatory protein PilR |
| Pili | ABZJ_03245 | -0.82 | 6.61E-05 | Protein PilH |
| Pili | ABZJ_03550 | -0.8 | 7.37E-03 | Pilin |
| Pili | ABZJ_00888 | -0.68 | 4.70E-03 | twitching mobility protein PilT |
| Iron uptake | ABZJ_01015 | -4.7 | 4.91E-04 | hypothetical protein |
| Iron uptake | ABZJ_01014 | -3.83 | 2.61E-04 | Iron siderophore receptor protein |
| Iron uptake | ABZJ_01012 | -3.13 | 1.58E-03 | Sigma-24 (FecI-like) |
| Iron uptake | ABZJ_01017 | -2.94 | 2.23E-03 | putative TonB-dependent receptor |
| Iron uptake | ABZJ_01018 | -2.52 | 6.38E-03 | Heme oxygenase HemO |
| Iron uptake | ABZJ_01013 | -2.23 | 7.58E-03 | Iron siderophore sensor protein |
| Iron uptake | ABZJ_01819 | -0.73 | 2.62E-04 | Fe-S cluster assembly scaffold IscU |
| Iron uptake | ABZJ_00312 | -0.61 | 5.16E-03 | uroporphyrinogen-III synthase |
| Capsule | ABZJ_00080 | -13.29 | 5.27E-11 | putative UDP-N-acetylglucosamine 2-epimerase legT |
| Capsule | ABZJ_00081 | -13.04 | 2.05E-11 | N-acetylneuraminate synthase legI |
| Capsule | ABZJ_00078 | -11.65 | 2.62E-10 | UDP-N-acetylglucosamine 4,6-dehydratase |
| Capsule | ABZJ_00079 | #NAME | 2.92E-11 | aminotransferase DegT |
| Capsule | ABZJ_00082 | #NAME | 2.39E-11 | alcohol dehydrogenase |
| Capsule | ABZJ_00083 | #NAME | 6.54E-04 | CMP-N-acetlyneuraminic acid synthetase neuA |
| potassium-transporting | ABZJ_02492 | -1.4 | 6.56E-04 | K..+..-transporting ATPase subunit B |
| potassium-transporting | ABZJ_02493 | -1.34 | 1.37E-04 | potassium-transporting ATPase subunit KdpA |
| potassium-transporting | ABZJ_02491 | -1.15 | 3.02E-03 | potassium-transporting ATPase subunit KdpC |
| potassium-transporting | ABZJ_02479 | -0.66 | 1.27E-03 | Putative potassium channel protein |
| chemotaxis | ABZJ_01242 | -0.95 | 2.44E-06 | chemotaxis protein |
| chemotaxis | ABZJ_03242 | -0.93 | 5.16E-06 | Gliding motility regulatory protein |
| Competence | ABZJ_00229 | -2.99 | 2.77E-07 | Competence protein ComM |
| desiccation | Novel00011 | -1.97 | 3.78E-03 | ATP-dependent Clp protease ATP-binding subunit ClpX |
| T2SS | ABZJ_02681 | -0.84 | 5.13E-05 | type II secretion system protein E epsE |
| ompW family | ABZJ_02425 | -0.79 | 3.19E-03 | ompW family proteins |
| Phospholipase | ABZJ_00067 | -0.66 | 2.71E-03 | phospholipase C, phosphocholine-specific |
| Note: #NAME means the readcounts value of the DST group is 0; padj: p-value after multiple hypothesis testing correction | | | | |

Table S9 Resistance genes upregulated in DST group

| **Category** | **gene_id** | **Log_2_(DST/NST)** | **padj** | **products** |
| --- | --- | --- | --- | --- |
| Aminoglycosides | ABZJ_01286 | Inf | 0.019241 | Aminoglycoside N(6&apos;)-acetyltransferase type 1 |
| Aminoglycosides | ABZJ_01287 | Inf | 0.007756 | Aminoglycoside N(6&apos;)-acetyltransferase type 1 |
| Aminoglycosides | ABZJ_p00004 | 10.02 | 2.60E-24 | Aminoglycoside 3&apos;-phosphotransferase |
| Chloramphenicol | ABZJ_01288 | Inf | 0.00004581 | Chloramphenicol acetyltransferase |
| Tetracyclines | ABZJ_02019 | 2.096 | 0.0039156 | TetR/AcrR family transcriptional regulator |
| Tetracyclines | ABZJ_00260 | 1.539 | 0.00082721 | Tetracycline resistance protein, class B |
| Tetracyclines | ABZJ_00261 | 1.415 | 0.0030971 | Tetracycline repressor protein class B from transposon Tn10 |
| Peptides | ABZJ_03224 | 1.263 | 2.26E-07 | peptide antibiotic transporter SbmA |
| beta lactams | ABZJ_02758 | 1.28 | 0.00058385 | Beta-lactamase |

Table S10 Resistance genes downregulated in DST group

| Category | gene_id | Log_2_（DST/NST） | padj | products |
| --- | --- | --- | --- | --- |
| chlorhexidine | ABZJ_02409 | -1.24 | 2.14E-06 | AceI family chlorhexidine efflux PACE |
| chloramphenicol | ABZJ_03118 | -1.12 | 3.08E-05 | chloramphenicol acetyltransferase CAT |
| Aminoglycosides | ABZJ_00199 | -0.82 | 1.49E-03 | GNAT family N-acetyltransferase |
